# Cushing Syndrome due to Adrenocortical Carcinoma During Pregnancy

**DOI:** 10.1210/jcemcr/luad118

**Published:** 2023-11-16

**Authors:** Jack Andrew Morris, Peter Campbell, Lily Xu, Anthony John O’Sullivan

**Affiliations:** Endocrinologist, Shoalhaven Hospital, Nowra, NSW, 2541, Australia; Breast Endocrine Surgeon, St. George Hospital, Kogarah, NSW, 2217, Australia; Department of Endocrinology, St. George Hospital, Kogarah, NSW, 2217, Australia; Department of Endocrinology, St. George Hospital, Kogarah, NSW, 2217, Australia; Professor of Medicine, St George and Sutherland Campus, School of Clinical Medicine, UNSW Sydney, Kogarah, NSW, 2217, Australia

**Keywords:** Cushing syndrome, pregnancy, adrenocortical carcinoma

## Abstract

Cushing syndrome resulting from adrenocortical carcinoma in pregnancy is exceedingly rare. There are no validated guidelines to establish a diagnosis or guide management in pregnancy. We provide a case of a 31-year-old woman presenting for management of diabetes in pregnancy who appeared cushingoid. She was subsequently diagnosed with ACTH-independent Cushing syndrome and experienced preterm labor at 33 weeks’ gestation, delivering a healthy infant. Four weeks postpartum, the patient underwent a left adrenalectomy and was subsequently diagnosed with adrenocortical carcinoma.

## Introduction

Adrenocortical carcinoma (ACC) is rare (incidence of 1–2 cases per million adults), more common in females, with a sex ratio of 4.2 (1). ACC diagnosed during pregnancy is exceedingly rare, and studies are limited to case reports (2). Cushing syndrome (CS) is uncommon during pregnancy because of reduced fertility associated with hypercortisolism and elevated androgens (2). Confirming the diagnosis of pathological hypercortisolism is also problematic because of physiological changes in the hypothalamic-pituitary-adrenal axis during pregnancy, which results in raised corticosteroid binding globulin and hypercortisolism. Investigations commonly used in the nonpregnant state are not well validated during pregnancy (3). Confirming the diagnosis of CS is imperative because of the adverse maternal and fetal outcomes experienced during pregnancy (4). We provide the case of a 31-year-old pregnant woman who presented for management of recently diagnosed gestational diabetes mellitus (GDM) and she was found to have features of CS on clinical examination. ACTH-independent CS and a 4-cm adrenal tumor were subsequently diagnosed. The pregnancy was complicated by premature rupture of membranes, and the patient delivered a female infant at 33 weeks’ gestation. Four weeks postpartum, the patient underwent a left adrenalectomy, which subsequently was identified as an ACC based on histopathology. Patient consent was obtained for publication of the case including clinical photography.

## Case Presentation

**Figure 1.**
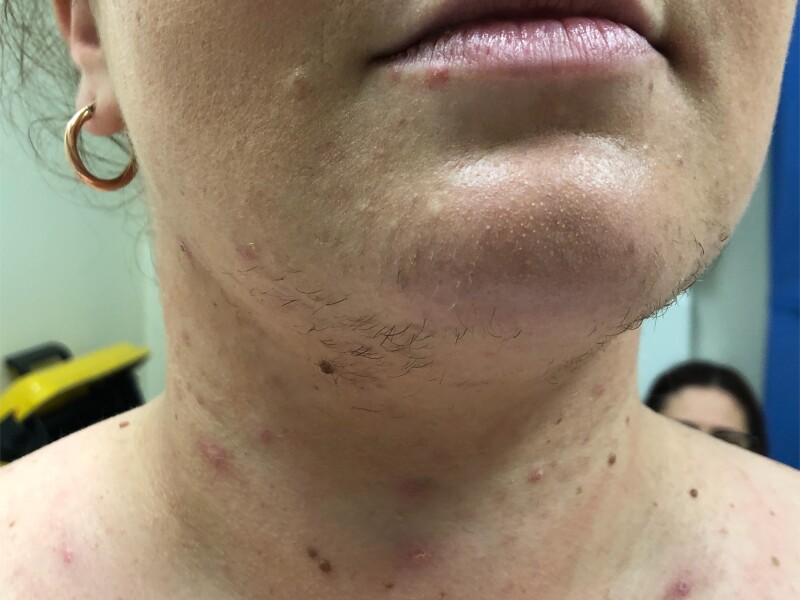
Androgenic facial hair.

**Figure 2.**
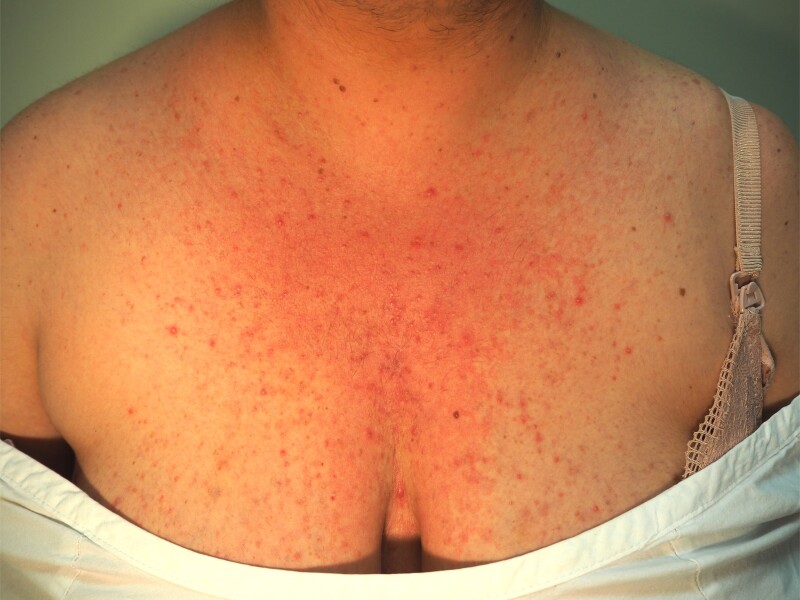
Chest acne.

**Figure 3.**
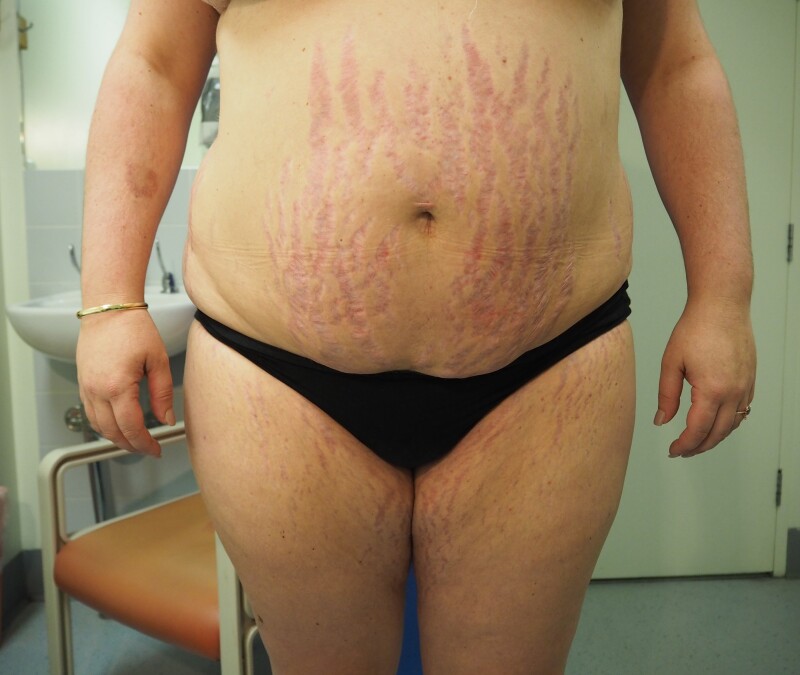
Violaceous abdominal striae.

A 31-year-old Caucasian woman (gravida 1, para 0) of 28 weeks’ gestation presented to the Endocrine Outpatient Department for the management of recently diagnosed GDM confirmed on 75-g oral glucose tolerance test according to Australian criteria (5) (fasting glucose = 5.3 mmol/L [95.5 mg/dL], 60 minutes’ glucose = 14.0 mmol/L [252 mg/dL], 120 minutes’ glucose = 8.8 mmol/L [159 mg/dL]). She had a history of Hashimoto hypothyroidism and was taking adequate thyroxine replacement therapy (TSH 0.3 mIU/L [0.3 IU/mL], reference range [RR], 0.31-2.75 mIU/L for 24-30 weeks’ gestation) and obesity (prepregnancy body mass index, 33.8 kg/m^2^). She worked as a beautician. In the initial consultation, she complained of developing coarse hair growth and acne over her face, neck, and chest during the pregnancy. There was no history of polycystic ovarian syndrome, prior glucocorticoid use, or adrenal disease. There was no family history of diabetes mellitus or adrenal disease. She had conceived naturally and her pregnancy was otherwise uncomplicated. An obstetric ultrasound at 22 weeks’ gestation demonstrated a singleton pregnancy with normal fetal morphology. On clinical examination, hirsutism with male facial and chest hair distribution, acne, and violaceous abdominal striae were confirmed (Figs. 1-3). There was no evidence of myopathy. She was hypertensive (blood pressure, 134/94 mm Hg) and clinically euthyroid.

A 24-hour urinary free cortisol (UFC) test was significantly elevated (968 nmol/24 hours [35 µg/24 hours], RR <166 nmol/24 hours [6 µg/24 hours]), as was testosterone (9.3 nmol/L [268 ng/dL], RR <2.0 nmol/L [58 ng/dL]), and androstenedione (20.2 nmol/L [579 ng/dL], RR 0.9-7.5 nmol/L [25.8-215 ng/dL]). ACTH was suppressed (3.6 ng/L; RR, 7.2-63.3 ng/L). Serum biochemistry, renal and liver function, metanephrines, normetanephrines, and aldosterone:renin ratio were within normal limits. Her dehydroepiandrosterone level was low (1.1 µmol/L [40.7 µg/dL]) compared with the age- and gender-specific RR (2.7-9.2 µmol/L [100-340 µg/dL]). Serially elevated 24-hour UFC and suppressed ACTH levels were used to confirmed the presence of ACTH-independent hypercortisolism. A dexamethasone suppression test and midnight salivary cortisol levels were not performed because of the lack of validation in pregnancy (2). An abdominal ultrasound demonstrated a left adrenal lesion measuring 28 × 22 × 23 mm. A magnetic resonance imaging (MRI) of the adrenal was planned, but was unable to be completed before delivery.

## Treatment

The patient's gestational diabetes was treated with metformin and subcutaneous basal glargine and bolus aspart insulin, which was subsequently well controlled. There was no evidence of fetal distress and the patient was otherwise stable. Endocrine surgery and obstetric medicine input was sought regarding further assessment and management of CS. Medical treatment was considered, although the patient ultimately delivered before it was able to be started.

At 32 weeks’ gestation, the patient experienced premature prelabor rupture of membranes and was admitted to the hospital. At 33 weeks’ gestation, she began to labor spontaneously and subsequently delivered a live female infant weighing 1879 g via forceps-assisted vaginal delivery. The newborn was admitted to the special care nursery for observation and received a short course of dexamethasone until adrenal suppression was excluded. There was no neonatal hypoglycemia and the infant demonstrated normal-appearing external genitalia, in contrast to previous case reports (6).

**Figure 4.**
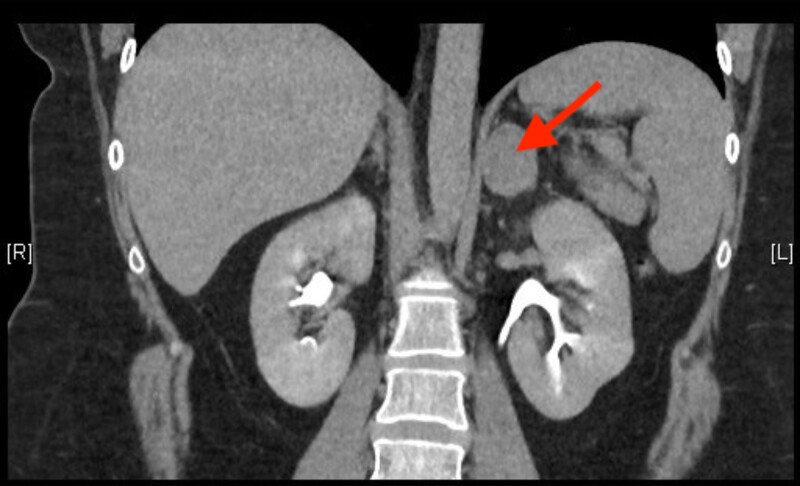
Coronal view of adrenal computed tomography scan demonstrating left adrenal lesion (red arrow).

An adrenal computed tomography scan performed postpartum confirmed the presence of a lesion measuring 3.2 cm in maximal diameter. The lesion was largely homogenous with scattered hypodense foci superiorly (27 Hounsfield units on a noncontrast computed tomography scan). The absolute and relative washout ratios were 66% and 42%, respectively, suggestive of an adenoma (Fig. 4). Repeat 24-hour UFC performed postpartum remained elevated (693 nmol/24 hour [25.1 µg/dL]) and ACTH remained suppressed (1.9 ng/L). Four weeks postpartum, the patient underwent a laparoscopic left adrenalectomy, which was uncomplicated. She completed a tapering course of prednisone that was discontinued after returning a normal short Synacthen test result 5 months postpartum.

## Outcome and Follow-up

Histopathology revealed a cortical lesion measuring 40 × 25 × 25 mm with high-grade nuclear features with pleomorphism, presence of giant cells, bizarre nuclei, myxoid deposits, and zones of tumor necrosis. Immunohistochemical staining was positive for inhibin, melanA. A Ki67 of 12% was identified in a few areas, but otherwise was very low (1%-2%) for most of the tumor (Fig. 3). There was no lymphovascular or capsular invasion. Overall, the features were consistent with an adrenocortical carcinoma (Weiss score: 3).

The patient underwent fluorodeoxyglucose-positron emission tomography, which did not demonstrate evidence of residual or metastatic disease. The case was discussed at a regional Endocrine-Oncology Multi-disciplinary meeting and, in consultation with the patient, a period of close surveillance was weighed against further surgery and chemo-radiotherapy. Because of the malignant nature of the lesion and strong preference to ensure microscopic clearance to protect against local or regional recurrence, the patient underwent further surgery consisting of an open laparotomy and resection of residual adrenal and adrenal bed tissue, which did not reveal any residual tumor or malignant cells. Over several months, her clinical signs of CS gradually improved.

## Discussion

CS resulting from ACC during pregnancy is rare. Adrenal lesions are responsible for approximately 50% of cases of CS during pregnancy, which is higher than CS in nonpregnant women (approximately 15%) (2). CS during pregnancy is associated with significant morbidity and mortality in both the fetus and mother (2). Comorbid hypertension, preeclampsia, weight gain, androgenization, and thrombogenic and catabolic effects of hypercortisolism are often observed. Diabetes mellitus is reported in 25% of pregnant women with CS (2-4). Clinical features, such as weight gain, fatigue, and peripheral edema often overlap with CS and a normal pregnancy, leading to a delayed or missed diagnosis, which may have been a contributing factor in this case.

Increased fetal morbidity and mortality has been widely reported, including premature labor (>50% of cases), growth restriction, sepsis, spontaneous abortion, and stillbirth (2-4). Adrenal suppression and virilization of genitalia in neonates have been reported; however, neither was observed in our case. Although the fetus may be partially protected from hypercortisolism by placental 11-beta-HSD2 and its conversion of cortisol to inactive cortisone, it is postulated that high levels of cortisol overwhelm enzyme capacity and mediate adverse effects (6, 7).

Confirming pathological hypercortisolism during pregnancy is problematic because of the physiological changes that occur within the hypothalamic-pituitary axes (3). Total cortisol levels are elevated 2-fold to 3-fold during pregnancy from an increase in corticosteroid-binding globulin and an increase in corticotropin secretion from both hypothalamic and placental sources (2). The diurnal pattern of cortisol secretion is maintained in pregnancy; however, suppression of cortisol by dexamethasone suppression test is reduced when measured against standard reference intervals derived from the nonpregnant population (2, 4). Although 24 hour UFC levels are elevated in the second and third trimesters, measurement is considered the most reliable method of diagnosing pathological hypercortisolism during pregnancy, with concentrations greater than 3 times the upper limit of normal considered diagnostic (8, 9). In our patient, an abdominal ultrasound was performed as the initial imaging modality because of its noninvasive nature and safety in pregnancy. The safety of MRI requires further evaluation; however, it has been suggested as the preferred imaging modality in pregnancy (7). In our case, an adrenal MRI scan was planned; however, the patient delivered before it could be completed.

There is no consensus on management of ACC during pregnancy and a paucity of data exists because of its rarity (7). Management should be individualized and based on the timing of diagnosis and severity. Compared with the nonpregnant population, patients diagnosed with ACC during pregnancy are reported to have a less favorable prognosis. One retrospective cohort study identified pregnant patients were more likely to be diagnosed at a later stage compared with nonpregnant women of childbearing age and had poorer overall survival (10).

In patients with CS resulting from adrenal lesions, definitive management with adrenalectomy in the second trimester is preferred over medical therapy (7). There are few published case reports of medical therapy for CS in pregnancy; however, metyrapone is often considered as first-line treatment (7, 8). Dosing can be adjusted with the aim of normalization of 24-h UFC. Careful monitoring of blood pressure is required because of the accumulation of mineralocorticoid precursors and risk of hypertension. Ketoconazole has also been used as a second-line agent (7). Thromboprophylaxis should also be considered because of the hypercoagulable states of pregnancy and CS. In our case, the patient was diagnosed in the third trimester and was unsuitable for surgery. Medical treatment was considered; however, the patient's GDM and blood pressure remained under good control and we did not commence medical therapy before delivery.

In this described case, the tumor was detected at an early stage and the patient remains disease free 3 years later after surgical treatment alone. Despite delivery of a premature infant at 33 weeks’ gestation, fetal outcomes were also favorable. The patient has since had an uncomplicated pregnancy, delivering a healthy infant. During her subsequent pregnancy, the patient did not have DGM and 24-hour UFC remained normal throughout gestation. Based on the outcomes in this case, we would recommend early multidisciplinary input and surgical intervention in patients diagnosed with ACC in pregnancy.

## Learning Points

- Cushing syndrome is rarely diagnosed during pregnancy.
- Confirming pathological hypercortisolism is problematic during pregnancy because of physiological changes to the hypothalamic-pituitary-adrenal axis.
- Because of the high prevalence of adverse outcomes, management of Cushing syndrome during pregnancy requires early multidisciplinary input from endocrinology, surgical, and obstetric teams.

## Data Availability

Data sharing is not applicable to this article as no datasets were generated or analyzed during the current study.

## Abbreviations

ACC: adrenocortical carcinoma
CS: Cushing syndrome
GDM: gestational diabetes mellius
MRI: magnetic resonance imaging
RR: reference range
UFC: urinary free cortisol
